# Sex- and age-dependent associations between waist-to-hip ratio and cognitive impairment: a cross-sectional study of rural adults aged 40 years and older in northwestern China

**DOI:** 10.3389/fnagi.2026.1835459

**Published:** 2026-08-13

**Authors:** Simeng Cui, Yu Zhang, Qiqi Qiang, Ziyu Liu, Peijie Liu, Liangjun Dang, Jin Wang, Wenhui Lu, Kang Huo, Yu Jiang, Chen Chen, Ling Gao, Shan Wei, Junlong Feng, Suixia Cao, Suhang Shang, Qiumin Qu, Meng Zhang

**Affiliations:** 1 Department of Neurology, The First Affiliated Hospital of Xi’an Jiaotong University, Xi’an, China; 2 Shaanxi Provincial Rehabilitation Hospital, Xi’an, China; 3 Department of Neurology, The Second Affiliated Hospital of Xi’an Jiaotong University, Xi’an, China; 4 Department of Encephalopathy, Huyi District Hospital of Traditional Chinese Medicine, Xi’an, China

**Keywords:** cognitive impairment, waist-to-hip ratio, age, sex, cross-sectional study, rural China

## Abstract

**Background:**

Obesity is a modifiable risk factor for cognitive impairment; however, body mass index fails to capture fat distribution. Waist-to-hip ratio (WHR), reflecting central adiposity, may better characterize obesity-related differences in cognitive impairment. Nevertheless, whether the association between WHR and cognitive impairment varies by sex and age remains unclear.

**Methods:**

This cross-sectional study included 1,792 adults aged ≥40 years from rural China. Cognitive impairment was defined using education-adjusted cutoffs on the Chinese Mini-Mental Status. WHR and clinical characteristics were collected by trained investigators. Multivariable logistic regression, restricted cubic splines, stratified analyses, and interaction analyses were performed.

**Results:**

In the total population, WHR was positively associated with the odds of cognitive impairment (OR = 1.245 per SD, 95% CI: 1.022–1.517, *P* = 0.029), with borderline evidence of nonlinearity (*P*_*overall*_ = 0.019, *P*_*nonlinear*_ = 0.090). In sex-stratified analyses, the association in females showed evidence of nonlinearity, with the estimated odds remaining relatively stable below a WHR of 0.88 and increasing at higher levels (*P*_*overall*_ = 0.014, *P*_*nonlinear*_ = 0.049), whereas no significant association was observed in males. Age-stratified analyses demonstrated a significant association in the middle-aged group (40–59 years, *P*_*overall*_ = 0.016, *P*_*nonlinear*_ = 0.143) but not in those ≥60 years. Further interaction analyses showed that age appeared to modify the association between WHR and cognitive impairment in males (OR = 0.462 per SD, 95% CI: 0.258–0.828, *P*_*interaction*_ = 0.010), but not in females. Specifically, the positive association between WHR and cognitive impairment was observed only in middle-aged males (OR = 1.652 per SD, 95% CI: 1.052–2.595, *P* = 0.029), but not in older males (OR = 0.804 per SD, 95% CI: 0.492–1.314, *P* = 0.384).

**Conclusion:**

Our findings show that WHR is associated with screening-defined cognitive impairment, with patterns that may differ by sex and age. Females exhibited a possible nonlinear association, whereas in males the association was mainly observed in midlife. These findings highlight central adiposity as an important correlate of cognitive impairment. Given the rural single-region sample, larger longitudinal studies with clinically adjudicated outcomes are warranted to assess generalizability and clarify temporal associations.

## 1 Introduction

Global population aging has contributed to a marked increase in the prevalence of cognitive impairment (Jia et al., 2020; Weidner, 2023), placing substantial burdens on families and society. Obesity is recognized as a potentially modifiable risk factor for cognitive impairment (Winblad et al., 2016). However, clinical studies based on body mass index (BMI) have reported inconsistent results regarding the association between obesity and cognitive impairment. Several studies have identified elevated BMI as a risk factor for cognitive impairment (Fan et al., 2019; Zhou et al., 2020), whereas others have proposed an “obesity paradox”, whereby higher BMI may act as a protective factor (Floud et al., 2020; Singh-Manoux et al., 2018). Such conflicting findings likely stem from fundamental limitations of BMI as a measure of obesity, which fails to distinguish between fat and lean mass or to capture fat distribution. In contrast, the waist-to-hip ratio (WHR) more accurately reflects central fat accumulation, a major anthropometric marker associated with metabolic syndrome and related pathologies. By better capturing the detrimental effects of visceral adiposity, WHR serves as a more reliable indicator of overall health status than waist circumference or BMI alone (Hsieh et al., 2003; Khan et al., 2023; Prillaman, 2023; Sweatt et al., 2024; Wang et al., 2002). Recent studies have examined central obesity, as reflected by WHR, in relation to cognitive impairment and have suggested a positive association (Hartanto et al., 2019; Luchsinger et al., 2012; Mina et al., 2023). However, the evidence remains limited and inconclusive.

A growing body of evidence suggests that the association between obesity, defined by BMI, and cognitive impairment is both sex- and age-dependent (Jacob et al., 2022; Livingston et al., 2024). For example, a recent systematic review and meta–analysis, including 14 studies and a total of 77,890 participants, confirmed that higher BMI in midlife is associated with an increased risk of all-cause dementia (Qu et al., 2020). Moreover, sex-dependent analyses have indicated that females may be more susceptible to the adverse effects of elevated BMI. However, despite the extensive focus on BMI, it remains unclear whether WHR – a measure that better captures central obesity – exhibits a similar sex- and age-dependent association with cognitive decline. This critical knowledge gap warrants further investigation.

Therefore, in this study, individuals aged 40 years and older from a village in the suburbs of Xi’an, northwestern China, were selected to assess the cross-sectional association between WHR and cognitive impairment, with a focus on sex- and age-specific differences.

## 2 Materials and methods

### 2.1 Data sources and study population

**FIGURE 1:**
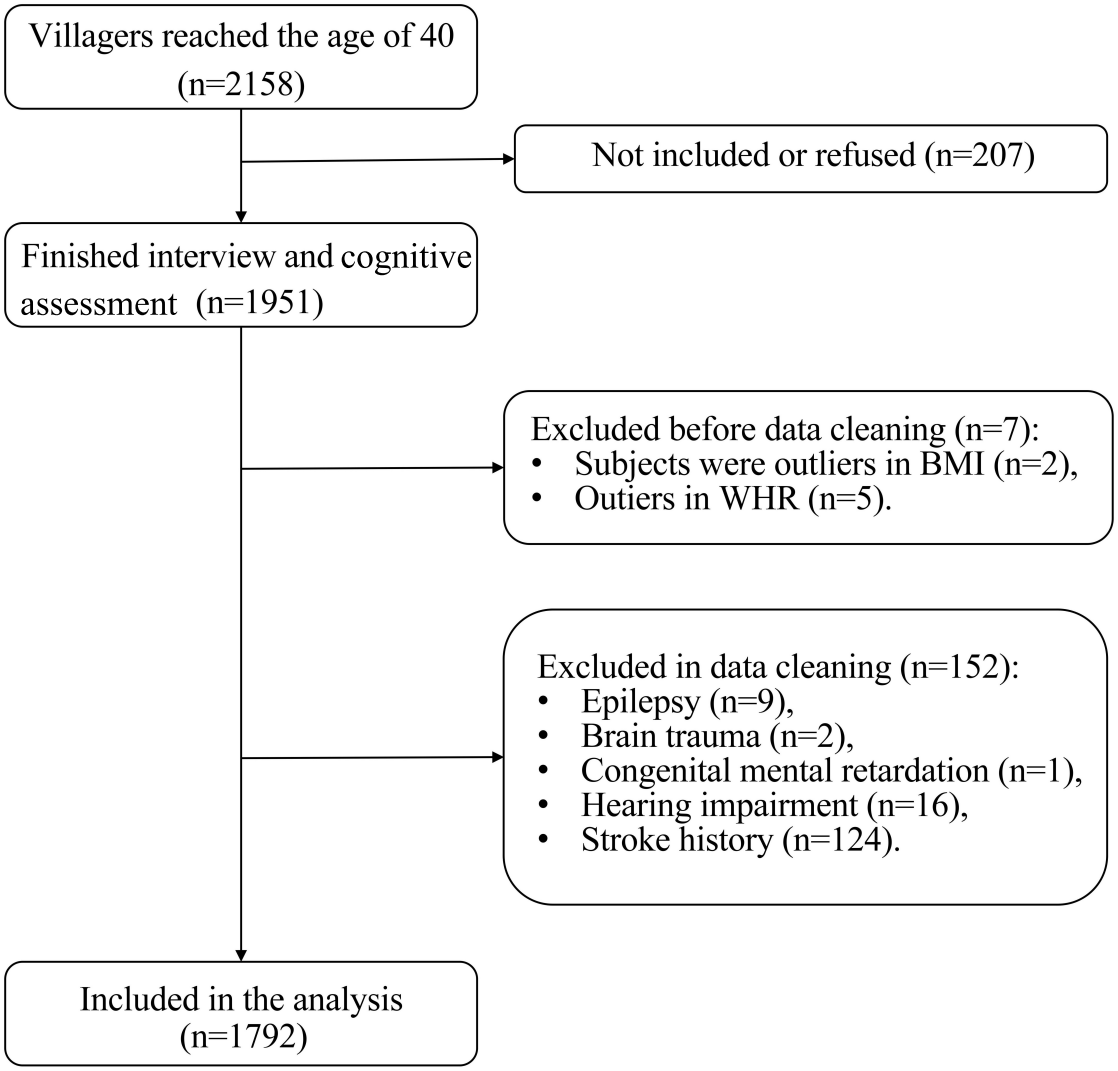
Flowchart of the study. BMI, body mass index; WHR, waist-to-hip ratio.

From October 2014 to March 2015, individuals from a village in the suburbs of Xi’an, northwestern China, were selected as the study population for cross-sectional analysis. The research procedure has been described in detail elsewhere (Shang et al., 2016). The screening process for participants is shown in Figure 1. Written informed consent was obtained from all participants, and the study was approved by the Medical Ethics Committee of the First Affiliated Hospital of Xi’an Jiaotong University.

The inclusion criteria were (1) permanent residents of Qubao village and Bitou village in Huyi District, Xi’an; (2) age ≥40 years at the time of the baseline survey; and (3) participants who provided written informed consent and completed the baseline survey. Exclusion criteria: (1) individuals with conditions that may affect cognitive function, such as stroke, chronic alcoholism, brain trauma, previous craniocerebral surgery, central nervous system tumor, intracranial infection, epilepsy, organic psychosis, schizophrenia, affective psychosis, congenital mental retardation or untreated hypothyroidism; (2) individuals with severe visual or hearing impairment that prevents completion of the cognitive assessment; (3) participants with severe cardiac, hepatic, or renal diseases, acute or terminal stages of a variety of chronic illnesses; and (4) participants with missing or abnormal WHR and BMI data (more than the mean ± 3SD).

### 2.2 Anthropometric measurements

Anthropometric indicators, including height, weight, waist circumference, and hip circumference, were measured by trained nurses according to standardized protocols (Kerwin et al., 2010). Participants were measured while standing, wearing light clothing, and without shoes where applicable. Waist circumference was measured 1 cm above the upper edge of the navel, and hip circumference was measured at the widest point between the waist and thigh. WHR was calculated as waist circumference divided by hip circumference. For 6 participants with missing anthropometric measurements, available self-reported data were used to calculate BMI and WHR, and 7 participants with outlying BMI or WHR values were excluded.

### 2.3 Cognitive assessment

Chinese Mini-Mental Status (CMMS) was used to assess global cognitive function. Cognitive impairment was defined as an CMMS score below the education-specific cutoff, specifically, ≤17 for illiterate participants, ≤20 for participants with secondary school education, and ≤24 for participants with junior high school education or above (Katzman et al., 1988).

### 2.4 Covariates assessment

Clinical characteristics, including demographic information, health-related behaviors, comorbidities, blood pressure measurements, and biochemical indicators, were collected. These variables included sex, age, years of education, education level, smoking status, alcohol consumption, physical activity, hypertension, diabetes, systolic blood pressure (SBP), diastolic blood pressure (DBP), fasting blood glucose (FBG), total cholesterol (TC), triglycerides (TG), high-density lipoprotein cholesterol (HDL-C), and low-density lipoprotein cholesterol (LDL-C). In the multivariable analyses, covariates included sex, age, education level, smoking status, alcohol consumption, physical inactivity, hypertension, FBG, TG, HDL-C, LDL-C, and BMI. Hypertension status was primarily determined from measured blood pressure, and self-reported medical history was used for the 12 participants without blood pressure measurements. Health-related behavioral information was collected through self-report. Regular exercise was defined as engaging in at least moderate-intensity physical activity, equivalent to brisk walking, at least three times per week for at least 30 min per session, or routinely performing moderate- or vigorous-intensity physical labor. Participants who did not meet this criterion were classified as physically inactive. Missing questionnaire-based covariate information was supplemented through telephone interviews within 3 months after the survey, and missing biochemical measurements were retested when possible. After follow-up interviews and retesting of biochemical measurements, missingness in the variables included in the final models was minimal. Complete-case analysis was used for multivariable modeling, and all primary fully adjusted models included 1,792 participants.

### 2.5 Statistical analysis

Data were analyzed by applying SPSS 26.0 software and R software (Version 4.4.1; R Core Team, 2024). Categorical variables were summarized as counts and percentages. Continuous variables were assessed for normality. Approximately normally distributed variables were summarized as mean ± standard deviation (SD), whereas skewed variables were summarized as median and interquartile range. Univariate analyses were performed using t-tests, analysis of variance, χ^2^ tests, and the Kruskal–Wallis rank sum test, depending on the purpose of the analysis and the type of data. All statistical tests were two-tailed with statistical significance set at 5% (α = 0.05).

Multivariate logistic regressions were used to estimate odds ratios (ORs) and 95% confidence intervals (CIs) for the association between WHR and cognitive impairment, adjusting for covariates. Cognitive impairment (yes/no) was the dependent variable, and WHR was the independent variable. The fully adjusted model included covariates selected based on previous evidence and between-group differences in univariate analyses, including sex, age, education level, smoking status, alcohol consumption, physical inactivity, hypertension, FBG, TG, LDL–C, HDL–C, and BMI. We additionally performed sensitivity analyses using sequential adjustment models. Model 1 adjusted for age, sex, education level, smoking status, alcohol consumption, and physical inactivity. Model 2 further adjusted for hypertension, FBG, TG, HDL-C, and LDL-C. Model 3 additionally adjusted for BMI. Multicollinearity among covariates was assessed using variance inflation factors. Multicollinearity was considered acceptable because all variance inflation factors were below 1.87. For stratified analyses by age and sex, stratification variables were not included as covariates in the corresponding subgroup models.

The key analytical steps were as follows: First, the association between WHR and cognitive impairment was examined in the total population using logistic regression. WHR was modeled using both restricted cubic splines (RCS) with three knots at the 25th, 50th, and 75th percentiles to assess potential nonlinear associations, and as a standardized continuous variable to estimate the linear association. In the RCS analyses, WHR was modeled on its original scale, with the median value (0.88) used as the reference point (OR = 1). For the linear analyses, WHR was standardized as a z score by subtracting the sample mean and dividing by the sample SD, and ORs were reported per 1-SD increase in WHR (SD = 0.06) to facilitate interpretation. Second, stratified analyses were performed by sex (females and males) and age group (40–59 vs. ≥60 years) to explore potential sex- and age-dependent patterns. Then, participants were divided into four subgroups: middle-aged females (females aged 40–59 years), older females (females aged ≥60 years), middle-aged males (males aged 40–59 years), and older males (males aged ≥60 years), and the same analyses were repeated within each stratum. Third, age-related effect modification was assessed separately in females and males by including WHR, age group (40–59 vs. ≥60 years), and their interaction term in logistic regression models. WHR was modeled both linearly per 1-SD increase, and using RCS. All models were adjusted for the same set of covariates as previously specified, and ORs with 95% confidence intervals were estimated across the WHR distribution.

## 3 Results

### 3.1 Demographics and clinical characteristics

A total of 1792 participants were included in the analysis, of whom 726 (40.5%) were male. Participants were aged between 40 and 85 years (mean 55.53 ± 9.92 years). The median WHR was 0.88 (0.84, 0.92). The median CMMS score was 27 (24, 29). In total, 230 participants (12.8%) were identified as having cognitive impairment. Participant characteristics according to cognitive impairment status are shown in Table 1.

**TABLE 1:** Characteristics of the study population.

Variables	Total population (*n* = 1792)	Cognitive impairment group (*n* = 230)	Normal cognitive group (*n* = 1562)	*P* value
Male [*n*, (%)]	726 (40.5)	86 (37.4)	640 (41.0)	0.302
Age [mean ± SD, years].	55.53 ± 9.92	61.52 ± 10.83	54.65 ± 9.47	<0.001
Years of education [Median (P25, P75), year]	7 (4, 8)	5 (0, 8)	8 (5, 9)	<0.001
Education level [*n*, (%)]				<0.001
Illiteracy	227 (12.7)	68 (29.6)	159 (10.2)	
Primary school	517 (28.9)	68 (29.6)	449 (28.7)	
Junior high school and above	1048 (58.5)	94 (40.9)	954 (61.1)	
Smoking status [*n*, (%)]	514 (28.7)	63 (27.4)	451 (28.9)	0.643
Alcohol consumption [*n*, (%)]	252 (14.1)	26 (11.3)	226 (14.5)	0.197
Physical inactivity [*n*, (%)]	272 (15.2)	46 (20.0)	226 (14.5)	0.029
Hypertension [*n*, (%)]	858 (47.9)	128 (55.7)	730 (46.7)	0.011
Diabetes [*n*, (%)]	220 (12.3)	44 (19.1)	176 (11.3)	0.001
BMI [mean ± SD, kg/m^2^]	25.29 ± 3.14	25.07 ± 3.15	25.37 ± 3.12	0.066
WHR [median (P25, P75), cm/cm]	0.88 (0.84, 0.92)	0.89 (0.84, 0.92)	0.88 (0.84, 0.92)	0.173
SBP [mean ± SD, mmHg]	131.71 ± 18.32	136.67 ± 20.09	130.98 ± 17.94	<0.001
DBP [mean ± SD, mmHg]	81.88 ± 10.16	83.18 ± 10.95	81.69 ± 10.02	0.051
FBG [median (P25, P75), mmol/L]	5.40 (5.07, 5.84)	5.42 (5.04, 6.02)	5.40 (5.07, 5.81)	0.315
TC [mean ± SD, mmol/L]	5.04 ± 1.00	5.10 ± 0.99	5.02 ± 1.00	0.130
TG [median (P25, P75), mmol/L]	1.44 (1.02, 2.01)	1.47 (1.02, 2.03)	1.43 (1.03, 2.00)	0.550
LDL–C [mean ± SD, mmol/L]	3.31 ± 0.90	3.34 ± 0.90	3.30 ± 0.90	0.375
HDL–C [mean ± SD, mmol/L]	1.40 ± 0.31	1.42 ± 0.30	1.39 ± 0.31	0.037
CMMS score [median (P25, P75)]	27 (24, 29)	19 (15, 23)	27 (25, 29)	<0.001

BMI: body mass index; WHR: waist-to-hip ratio; SBP: systolic blood pressure; DBP: diastolic blood pressure; FBG: fasting blood glucose; TC: total cholesterol; TG: triglycerides; LDL–C: low-density lipoprotein cholesterol; HDL–C: high–density lipoprotein cholesterol; CMMS: Chinese Mini-Mental Status.

### 3.2 Association between WHR and cognitive impairment in the total population

**FIGURE 2:**
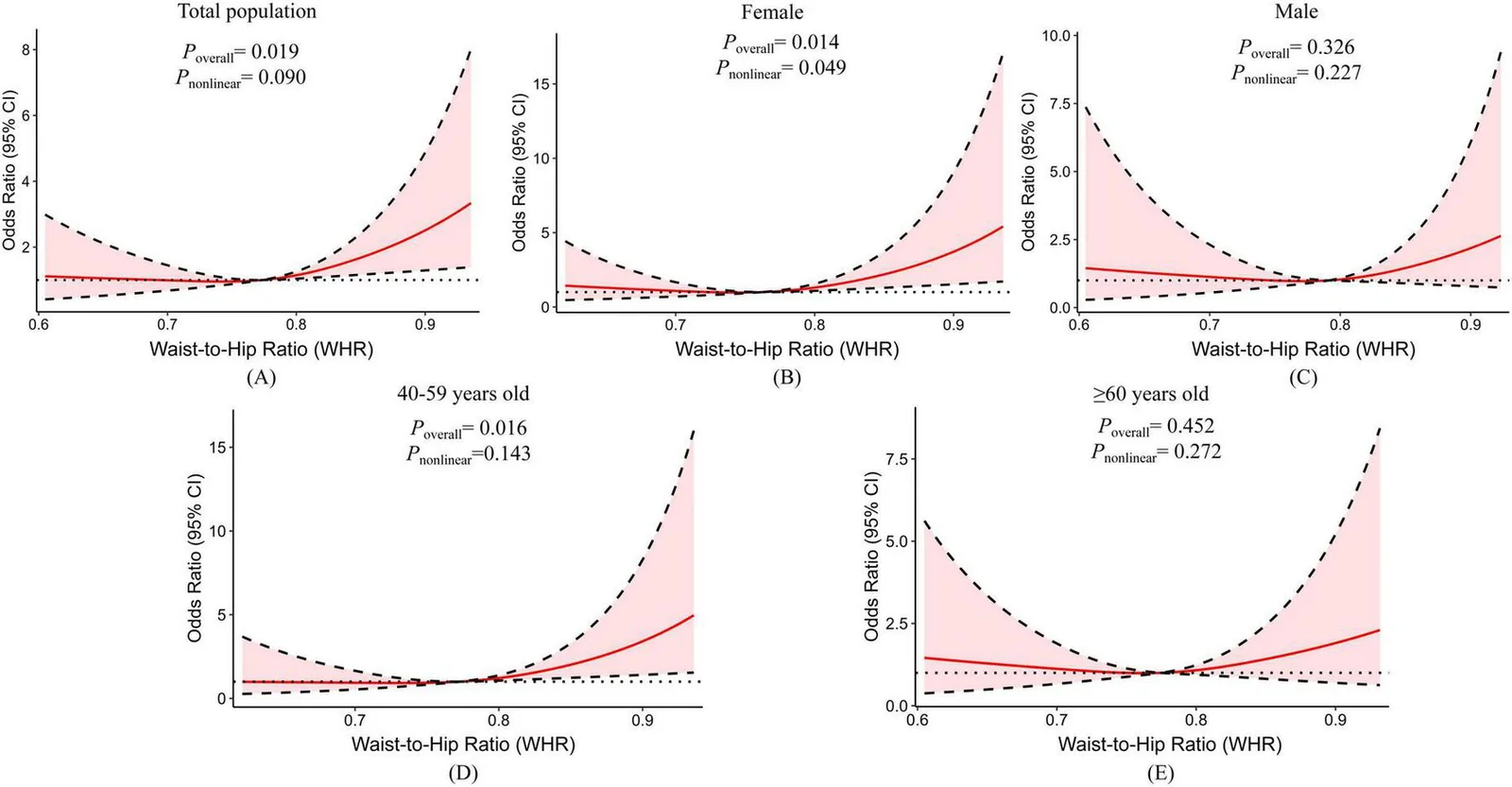
Estimated odds ratios for cognitive impairment across the WHR distribution in the total population **(A)**, females **(B)**, males **(C)**, the middle-aged (40–59 years, **D**), and older (≥60 years, **E**) age groups. In the logistic regression model, the WHR was modeled using a restricted cubic spline with three knots placed at the 25th, 50th, and 75th percentiles of the WHR distribution. The reference value was set at WHR = 0.88. Curves represent adjusted odds ratios for cognitive impairment estimated using restricted cubic spline models, with shaded areas indicating 95% confidence intervals. Models were adjusted for age, sex, education level, smoking status, alcohol consumption, physical inactivity, hypertension, FBG, TG, LDL–C, HDL–C, and BMI. Stratification variables were not included as covariates in the corresponding subgroup models.

WHR was positively associated with the odds of cognitive impairment in the total population (Table 2, OR = 1.245 per SD, 95% CI: 1.022–1.517, *P* = 0.029), while the test for nonlinearity showed borderline evidence (Figure 2A, *P*_*overall*_ = 0.019, *P*_*nonlinear*_ = 0.090).

**TABLE 2:** Multivariate logistic regression of the association between standardized WHR and cognitive impairment in the total population and subgroup analyses stratified by sex and age.

Group	N	B	Standard error	Wald	OR (95% CI), per SD	*P* value
Total population	1792	0.219	0.101	4.749	1.245 (1.022, 1.517)	0.029
Females	1066	0.269	0.129	4.355	1.308 (1.016, 1.684)	0.037
Males	726	0.139	0.166	0.704	1.149 (0.830, 1.591)	0.402
40–59 years	1193	0.341	0.144	5.644	1.406 (1.061, 1.863)	0.018
≥60 years	599	0.086	0.144	0.357	1.090 (0.822, 1.446)	0.550
Middle-aged females	722	0.271	0.190	2.028	1.311 (0.903, 1.903)	0.154
Older females	344	0.266	0.179	2.213	1.305 (0.919, 1.853)	0.137
Middle-aged males	471	0.502	0.230	4.748	1.652 (1.052, 2.595)	0.029
Older males	255	-0.218	0.250	0.757	0.804 (0.492, 1.314)	0.384

In logistic regression models, WHR was standardized as a z score using the sample mean and SD. ORs are presented per 1-SD increase in WHR (SD = 0.06). The standardized WHR was modeled as a continuous predictor to assess its linear association with the odds of cognitive impairment in the total population and in subgroups defined by sex (females and males), age (40–59 years and ≥60 years), and their combinations (middle-aged females, older females, middle-aged males, and older males). All models were adjusted for age, sex, education level, smoking status, alcohol consumption, physical inactivity, hypertension, FBG, TG, LDL-C, HDL-C, and BMI. Stratification variables were not included as covariates in the corresponding subgroup models.

### 3.3 Sex-dependent association between WHR and cognitive impairment

Participants were stratified into female and male subgroups, and the association between WHR and cognitive impairment was examined separately in females and males. Among females, WHR was positively associated with the odds of cognitive impairment (Table 2, OR = 1.308 per SD, 95% CI: 1.016–1.684, *P* = 0.037). The spline curve was broadly similar to that observed in the total population, but evidence of nonlinearity was stronger among females (Figure 2B, *P*_*overall*_ = 0.014, *P*_*nonlinear*_ = 0.049). Specifically, using 0.88 as the reference value (OR = 1), the spline curve suggested relatively stable estimated odds below this value and increasing estimated odds at higher WHR levels. However, among males, WHR was not significantly associated with cognitive impairment (Table 2, OR = 1.149 per SD, 95% CI: 0.830–1.591, *P* = 0.402; Figure 2C, *P*_*overall*_ = 0.326, *P*_*nonlinear*_ = 0.227).

### 3.4 Age-dependent association between WHR and cognitive impairment

To examine the age-dependent association between WHR and cognitive impairment, the total population was divided into middle-aged (40–59 years) and older (≥60 years) groups. In the 40–59-year-old subgroup, WHR was positively associated with the odds of cognitive impairment (Table 2, OR = 1.406 per SD, 95% CI: 1.061–1.863, *P* = 0.018), with no statistically significant evidence of nonlinearity (Figure 2D, *P*_*overall*_ = 0.016, *P*_*nonlinear*_ = 0.143); however, in the ≥60-year-old subgroup, no significant association between WHR and cognitive impairment was observed (Table 2, OR = 1.090 per SD, 95% CI: 0.822–1.446, *P* = 0.550; Figure 2E, *P*_*overall*_ = 0.452, *P*_*nonlinear*_ = 0.272).

### 3.5 Interaction effect of age on the association between WHR and cognitive impairment

These findings suggest that the association between WHR and cognitive impairment may vary by age and sex. To further explore this interaction, the total population was stratified into four subgroups based on sex and age: females aged 40–59 years, females aged ≥60 years, males aged 40–59 years, and males aged ≥60 years.

**FIGURE 3:**
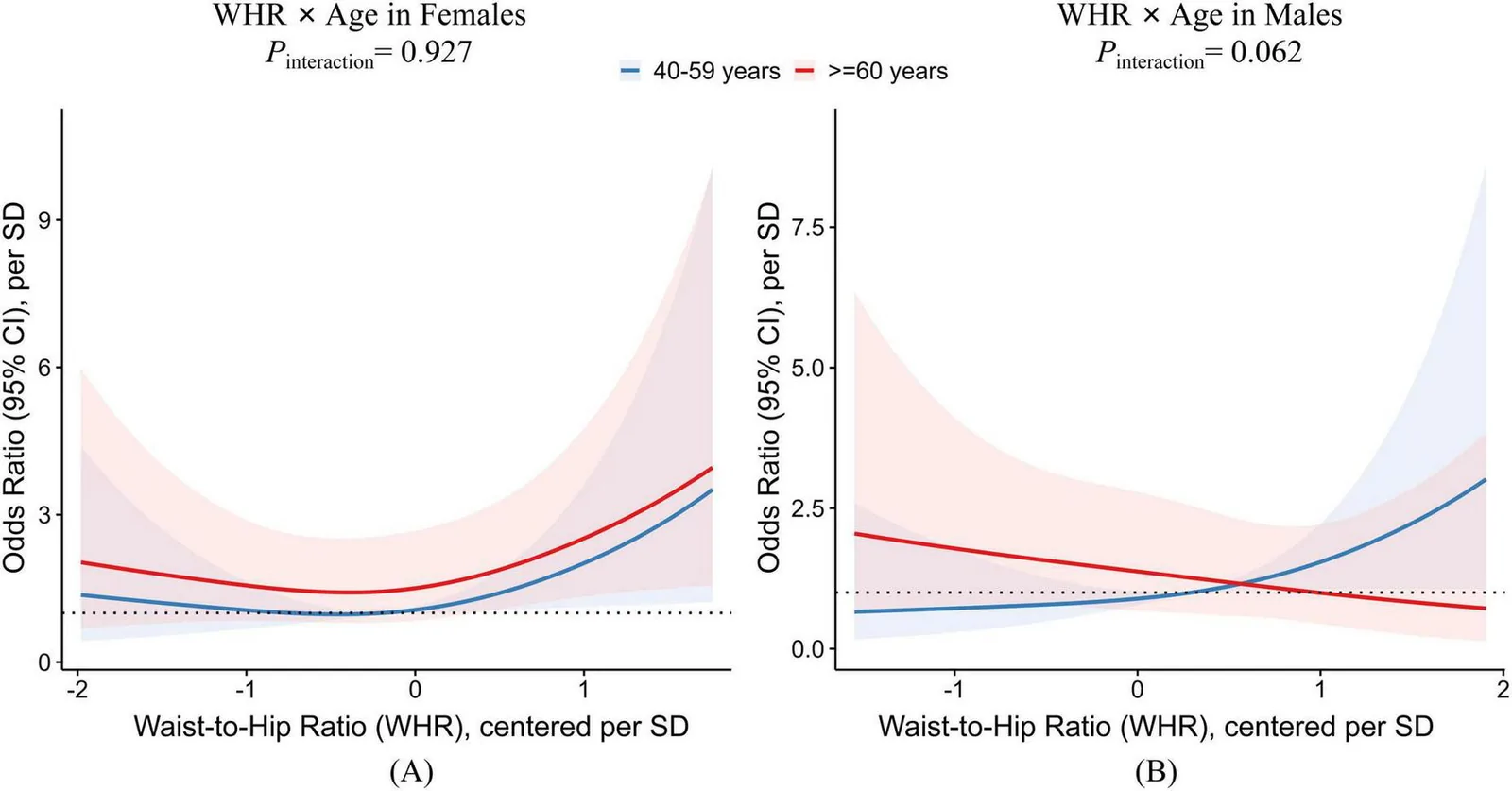
Age-related effect modification of the association between WHR and cognitive impairment in females **(A)** and males **(B)**. Two-way interaction terms between WHR and age group (40–59 years vs. ≥60 years) were incorporated into sex-stratified logistic regression models. ORs were reported per 1-SD increase in WHR (SD = 0.06). The blue and red curves represent participants aged 40–59 years and ≥60 years, respectively. All models were adjusted for education level, smoking status, alcohol consumption, physical inactivity, hypertension, FBG, TG, LDL-C, HDL-C, and BMI.

Among females, the RCS curves for the two age groups ran nearly parallel, with no evidence of effect modification by age (RCS WHR × age group *P*_*interaction*_ = 0.927; Figure 3A). Consistent with this visual pattern, the linear interaction term was also non-significant (continuous WHR × age group *P*_*interaction*_ = 0.790; OR = 0.939 per SD, 95% CI: 0.590–1.493; Table 3), indicating that the association between WHR and cognitive impairment did not differ meaningfully between the middle-aged and older females.

**TABLE 3:** Interaction effect of age on the association between WHR and cognitive impairment, stratified by sex.

Variable	B	standard error	Wald	OR (95% CI), per SD	*P* value
Female (n = 1066)					
Age (40–59 years vs. ≥60 years)	0.350	0.239	2.140	1.419 (0.888, 2.269)	0.144
WHR (Standarized)	0.293	0.182	2.605	1.340 (0.939, 1.913)	0.107
WHR × Age (Interaction term)	−0.063	0.237	0.071	0.939 (0.590, 1.493)	0.790
Male (n = 726)					
Age (40–59 years vs. ≥60 years)	0.327	0.295	1.229	1.387 (0.778, 2.475)	0.268
WHR (Standarized)	0.484	0.213	5.149	1.622 (1.068, 2.464)	0.023
WHR × Age (Interaction term)	−0.771	0.297	6.723	0.462 (0.258, 0.828)	0.010

Interaction analyses were performed separately in females and males. WHR, age group (40–59 vs. ≥60 years), and their interaction term (WHR × age group) were simultaneously included in the logistic regression models to assess potential effect modification by age. WHR was standardized as a z score using the sample mean and SD. ORs are presented per 1-SD increase in WHR (SD = 0.06). All models were adjusted for education level, smoking status, alcohol consumption, physical inactivity, hypertension, FBG, TG, LDL-C, HDL-C, and BMI. Stratification variables were not included as covariates in the corresponding subgroup models.

Among males, the RCS curves for the two age groups diverged in opposite directions, suggesting potential age-dependent heterogeneity in the association between WHR and cognitive impairment. The RCS interaction showed borderline evidence of interaction (RCS WHR × age group *P*_*interaction*_ = 0.062; Figure 3B). Given the absence of strong evidence for nonlinearity in the overall WHR–cognitive impairment association among males, the pre-specified linear interaction model was regarded as the summary of age-related heterogeneity and demonstrated a significant WHR × age group interaction (continuous WHR × age group *P*_*interaction*_ = 0.010; OR = 0.462 per SD, 95% CI: 0.258–0.828; Table 3). Specifically, WHR was positively and significantly associated with the odds of cognitive impairment in males aged 40–59 years (OR = 1.652 per SD, 95% CI: 1.052–2.595, *P* = 0.029, Table 2), whereas no significant association was observed in males aged ≥60 years (OR = 0.804 per SD, 95% CI: 0.492–1.314, *P* = 0.384, Table 2). These findings suggest age-related heterogeneity in males, with the positive association observed between WHR and cognitive impairment in middle-aged but not older males.

### 3.6 Sensitivity analyses

Sensitivity analyses using sequential adjustment models are presented in Supplementary Table 1. In the total population, the OR per 1-SD increase in WHR increased modestly from 1.152 (95% CI: 0.963–1.378) in Model 1 to 1.245 (95% CI: 1.022–1.517) in Model 3. Among females and participants aged 40–59 years, the direction and magnitude of associations remained broadly similar across the sequential models, whereas no significant associations were observed among males or participants aged ≥60 years. In the sex-by-age subgroup analyses, the ORs among middle-aged males increased from 1.266 (95% CI: 0.861–1.862) in Model 1 to 1.652 (95% CI: 1.052–2.595) in Model 3, whereas the inverse association among older males was progressively attenuated (OR = 0.599, 95% CI: 0.389–0.922 in Model 1 vs. OR = 0.804, 95% CI: 0.492–1.314 in Model 3). The corresponding RCS analyses yielded similar results. Across Models 1–3, evidence of nonlinearity was limited to females (*P*_nonlinear_ = 0.047, 0.041, and 0.049), whereas no significant nonlinear associations were observed in the other subgroups.

In sequential interaction analyses (Supplementary Table 2), no significant WHR × age group interaction was observed among females across Models 1–3. In contrast, the linear WHR × age group interaction remained statistically significant among males after sequential adjustment (continuous WHR × age group *P*_interaction_ = 0.017, 0.014, and 0.010), with borderline RCS interaction results in Models 2–3 (RCS WHR × age group *P*_interaction_ = 0.082 and 0.062).

## 4 Discussion

Overall, our findings suggest distinct age- and sex-specific patterns in the association between WHR and screening-defined cognitive impairment. Among females, WHR showed evidence of nonlinear association with cognitive impairment, with higher odds observed when WHR exceeded approximately 0.88; this pattern was similar across both 40–59-year and ≥60-year groups in females. In contrast, for males, age exhibited a potential interactive effect – specifically, a positive association between WHR and cognitive impairment was evident only in the middle-aged group, whereas no such association was detected among older males.

These findings are broadly consistent with existing hypotheses linking central adiposity to biological processes involved in cognitive decline and further suggest that the WHR–cognitive impairment association varies by sex and age. Specifically, this association appeared more evident among females and middle-aged males, highlighting a distinct demographic heterogeneity. Furthermore, exploratory nonlinear analysis in females suggested a threshold-like pattern, where higher WHR values were associated with greater odds of cognitive impairment above approximately 0.88. This value closely aligns with the WHO abdominal obesity cut-off (World Health Organization, 2011), a point above which metabolic and vascular disturbances related to visceral adiposity may become more prominent (Debette et al., 2014; Han et al., 2021; Hosseini et al., 2024; Ou et al., 2015; Widjaja et al., 2023; Willette and Kapogiannis, 2015), and are hypothesized to adversely affect brain structure and function (Costache et al., 2023; Jagust et al., 2005). Although this nonlinear pattern should be interpreted cautiously and viewed as exploratory, it suggests that WHR may be a useful anthropometric correlate for future studies of cognitive impairment, beyond its conventional role in metabolic risk assessment.

Our findings extend existing evidence by demonstrating a distinct sex- and age-specific pattern in the association between WHR and cognitive impairment, highlighting differential vulnerability across life stages. In females, hormonal changes around menopause, particularly the sharp decline in estrogen levels (Hogervorst et al., 2023), promote visceral fat accumulation (Nishimura et al., 2014) and may contribute to inflammation and Aβ deposition that have been hypothesized to be correlated with elevated cognitive vulnerability(Christensen and Pike, 2017). Overall, these findings are broadly in line with earlier studies, though not entirely consistent, which suggest greater susceptibility to obesity-related cognitive decline for females (Liu et al., 2021; Shang et al., 2023). This biological susceptibility may be compounded by sociocultural factors (Hogervorst et al., 2023), such as lower educational attainment, potentially amplifying the cognitive impact of abdominal obesity. In males, the positive association was restricted to midlife, consistent with the concept that midlife represents a critical window during which obesity shows stronger associations with cognitive health (Anstey et al., 2011; Livingston et al., 2024). Compared with the abrupt decline in estrogen following menopause, age-related declines in testosterone among males are generally more gradual (Hogervorst et al., 2023), which may partly explain the absence of a significant association in later life. Alternatively, survival bias may also partly explain this pattern, as males with higher WHR and greater susceptibility to adverse health outcomes may be less likely to survive into older age, resulting in a relatively healthier surviving cohort (Smith et al., 2011). These findings underscore the importance of considering sex- and age-specific factors when examining the association between central obesity and cognitive health.

A methodological strength of this study is the use of WHR as the primary adiposity indicator. Compared with BMI, WHR better captures central obesity and its relevance to cognitive health. Although BMI is widely used in clinical practice, its inability to distinguish between fat and lean mass limits its utility in capturing obesity-related metabolic risk and its association with cognitive function (Prillaman, 2023; Sweatt et al., 2024; Wang et al., 2002). This distinction is also important for interpreting the adjusted models. Because BMI and metabolic factors may partly lie on the pathway linking central adiposity to cognitive outcomes, the fully adjusted model should be interpreted as estimating the association of WHR independent of overall adiposity and selected metabolic factors, rather than the total association of central adiposity with cognitive impairment. Waist circumference offers a better measure of abdominal fat but does not account for body shape differences related to height. In contrast, WHR more precisely captures abdominal fat distribution, which is associated with inflammation and cardiovascular risk factors (de Koning et al., 2007; Xu et al., 2024), and has therefore been proposed as a more informative indicator in studies of cognitive function (Ntlholang et al., 2018). Previous studies have generally reported that higher WHR is associated with greater odds of cognitive impairment (Hartanto et al., 2019; Luchsinger et al., 2012; Mina et al., 2023). Our findings broadly align with this evidence, particularly in females and middle-aged adults, supporting that WHR may be an anthropometric correlate associated with greater odds of screening-defined cognitive impairment in these groups. However, no significant association was observed in older males, which may reflect differences in population characteristics or study design and suggests potential age- and sex-dependent variations that warrant further investigation. These findings help characterize subgroup differences in the WHR-cognitive impairment association and provide insights for future longitudinal research evaluating the potential value of WHR for sex- and age-specific risk assessment.

Despite considerable effort to obtain reliable data, some limitations remain. First, as a cross-sectional study, this study cannot establish temporal or causal relationships between WHR and cognitive impairment. Thus, future prospective cohort studies are warranted to confirm our findings. Second, although we adjusted for a wide range of covariates and excluded participants with a history of stroke, residual confounding cannot be entirely excluded. For example, information on APOE ε4 status, coronary heart disease, medication use, depression, and socioeconomic factors was not available in the current study. Third, the study participants were from rural areas in Northwest China, which may limit the generalizability of the findings. Additionally, certain stratified analyses yielded borderline significant results, accompanied by minor discrepancies between the linear and RCS models and wider confidence intervals at the tails of the WHR distribution in the RCS analyses. These findings likely reflect the reduced amount of information available after stratification. Therefore, these subgroup-specific findings should be interpreted with caution and require validation in larger and more diverse populations to confirm the observed age- and sex-specific patterns. Finally, CMMS-based screening cannot distinguish specific subtypes of cognitive impairment, such as Alzheimer’s disease or vascular dementia. However, obesity has been identified as a shared risk factor for both Alzheimer’s disease and vascular dementia, the two most common dementia subtypes (Beydoun et al., 2008). Therefore, although CMMS cannot distinguish specific etiologies of cognitive impairment, it remains appropriate for evaluating population-level associations between WHR and global cognitive impairment.

## 5 Conclusion

In this cross-sectional study of adults aged 40 years and older from a rural region of northwestern China, higher WHR was associated with screening-defined cognitive impairment, with patterns that appeared to differ by sex and age. Among females, spline analyses suggested a potentially nonlinear association. Among males, a positive linear association was observed in middle-aged males but not in older males. These findings suggest that WHR may be a useful anthropometric correlate for future studies of cognitive impairment, while highlighting the importance of considering both age and sex. Given the rural single-region sample, further longitudinal studies in larger and more diverse populations, with clinically adjudicated cognitive outcomes, are needed to assess generalizability, clarify temporal associations, and explore the potential mechanisms linking central adiposity to cognitive function.

## Data Availability

The raw data supporting the conclusions of this article will be made available by the corresponding author on reasonable request.
