# Supplementary material for: Sex- and age-dependent associations between waist-to-hip ratio and cognitive impairment: a cross-sectional study of rural adults aged 40 years and older in northwestern China
Source: Front Aging Neurosci. 2026 Aug 13;18:1835459. doi: 10.3389/fnagi.2026.1835459 (PMC13518583; doi:10.3389/fnagi.2026.1835459)
Supplement: Supplementary file 1 [file Table_1.docx]

**Supplementary Table S1. Sequential adjustment analyses of the association between waist-to-hip ratio and cognitive impairment**

| Subgroup | N | Model | OR (95% CI), per SD | *P* value | RCS *P*_overall_ | *RCS P*_nonlinear_ |
| --- | --- | --- | --- | --- | --- | --- |
| Total population | 1792 | Model 1 | 1.152 (0.963, 1.378) | 0.122 | 0.047 | 0.061 |
|  |  | Model 2 | 1.156 (0.960, 1.393) | 0.127 | 0.053 | 0.068 |
|  |  | Model 3 | 1.245 (1.022, 1.517) | 0.029 | 0.019 | 0.090 |
| Female | 1066 | Model 1 | 1.284 (1.019, 1.618) | 0.034 | 0.012 | 0.047 |
|  |  | Model 2 | 1.255 (0.987, 1.594) | 0.064 | 0.019 | 0.041 |
|  |  | Model 3 | 1.308 (1.016, 1.684) | 0.037 | 0.014 | 0.049 |
| Male | 726 | Model 1 | 0.929 (0.697, 1.239) | 0.616 | 0.321 | 0.157 |
|  |  | Model 2 | 1.002 (0.739, 1.358) | 0.992 | 0.446 | 0.204 |
|  |  | Model 3 | 1.149 (0.830, 1.591) | 0.402 | 0.326 | 0.227 |
| 40-59 years | 1193 | Model 1 | 1.321 (1.024, 1.705) | 0.032 | 0.024 | 0.123 |
|  |  | Model 2 | 1.352 (1.032, 1.771) | 0.028 | 0.022 | 0.128 |
|  |  | Model 3 | 1.406 (1.061, 1.863) | 0.018 | 0.016 | 0.143 |
| ≥60 years | 599 | Model 1 | 0.939 (0.731, 1.207) | 0.623 | 0.456 | 0.252 |
|  |  | Model 2 | 0.919 (0.709, 1.190) | 0.521 | 0.382 | 0.223 |
|  |  | Model 3 | 1.090 (0.822, 1.446) | 0.550 | 0.452 | 0.272 |
| Middle-aged females | 722 | Model 1 | 1.312 (0.932, 1.847) | 0.120 | 0.059 | 0.092 |
|  |  | Model 2 | 1.290 (0.897, 1.856) | 0.169 | 0.072 | 0.080 |
|  |  | Model 3 | 1.311 (0.903, 1.903) | 0.154 | 0.070 | 0.085 |
| Older females | 344 | Model 1 | 1.181 (0.863, 1.615) | 0.298 | 0.185 | 0.133 |
|  |  | Model 2 | 1.115 (0.809, 1.536) | 0.506 | 0.307 | 0.167 |
|  |  | Model 3 | 1.305 (0.919, 1.853) | 0.137 | 0.148 | 0.216 |
| Middle-aged males | 471 | Model 1 | 1.266 (0.861, 1.862) | 0.231 | 0.304 | 0.372 |
|  |  | Model 2 | 1.518 (0.989, 2.328) | 0.056 | 0.112 | 0.490 |
|  |  | Model 3 | 1.652 (1.052, 2.595) | 0.029 | 0.069 | 0.547 |
| Older males | 255 | Model 1 | 0.599 (0.389, 0.922) | 0.020 | 0.064 | 0.931 |
|  |  | Model 2 | 0.653 (0.414, 1.029) | 0.066 | 0.186 | 0.991 |
|  |  | Model 3 | 0.804 (0.492, 1.314) | 0.384 | 0.685 | 0.988 |

Values are presented as odds ratios (ORs) with 95% confidence intervals (CIs) and *P* values per 1-SD increase in waist-to-hip ratio (WHR; SD = 0.06). Restricted cubic spline analyses are reported using *P*_overall_ and *P*_nonlinear_. Model 1 adjusted for age, sex, education level, smoking status, alcohol consumption, and physical inactivity, where applicable. Model 2 further adjusted for hypertension and metabolic covariates, including FBG, TG, LDL-C, and HDL-C. Model 3 further adjusted for BMI. Stratification variables were not included as covariates in the corresponding subgroup models.
