# Supplementary material for: Sex- and age-dependent associations between waist-to-hip ratio and cognitive impairment: a cross-sectional study of rural adults aged 40 years and older in northwestern China
Source: Front Aging Neurosci. 2026 Aug 13;18:1835459. doi: 10.3389/fnagi.2026.1835459 (PMC13518583; doi:10.3389/fnagi.2026.1835459)
Supplement: Supplementary file 2 [file Table_2.docx]

**Supplementary Table S2. Sequential adjustment analyses for the interaction between WHR and age group, stratified by sex**

| **Sex** | N | Model | OR (95% CI), per SD | Continuous  WHR × age  *P*_interaction_ | RCS  WHR × age  *P*_interaction_ |
| --- | --- | --- | --- | --- | --- |
| Female | 1066 | Model 1 | 0.903 (0.570–1.433) | 0.666 | 0.871 |
|  |  | Model 2 | 0.918 (0.576–1.463) | 0.718 | 0.884 |
|  |  | Model 3 | 0.939 (0.590–1.493) | 0.790 | 0.927 |
| Male | 726 | Model 1 | 0.495 (0.278–0.881) | 0.017 | 0.103 |
|  |  | Model 2 | 0.485 (0.272–0.865) | 0.014 | 0.082 |
|  |  | Model 3 | 0.462 (0.258–0.828) | 0.010 | 0.062 |

Interaction analyses were performed separately in females and males. Linear models included WHR, age group (40–59 vs. ≥60 years), and the WHR × age group interaction term, with ORs reported for the interaction term per 1-SD increase in WHR (SD = 0.06). RCS models modeled WHR using restricted cubic splines and tested the WHR × age group interaction. Model 1 adjusted for education level, smoking status, alcohol consumption, and physical inactivity; Model 2 further adjusted for hypertension, FBG, TG, HDL-C, and LDL-C; Model 3 further adjusted for BMI.
